# Inverse hysteresis effects in a real and virtual environment

**DOI:** 10.3389/fspor.2026.1734971

**Published:** 2026-02-25

**Authors:** Christoph Schütz

**Affiliations:** 1 Faculty of Psychology and Sports Science, Bielefeld University, Bielefeld, Germany; 2 Center for Cognitive Interaction Technology, Bielefeld University, Bielefeld, Germany

**Keywords:** full-body movement, hysteresis, motor planning, posture selection, real environment, virtual environment, visual feedback

## Abstract

In sequential, repetitive motor tasks such as opening a stack of drawers, we tend to reuse prior motor plans to reduce cognitive load. This persistence, termed motor hysteresis, has been demonstrated in discrete reaching movements but not in discrete full-body movements. If hysteresis reflects a general principle of cost reduction, it should also manifest in full-body movements. To test this, we asked participants to perform a discrete full-body posture selection task: passing through apertures of varying widths. Width was varied in ordered sequences to induce hysteresis and body rotation was measured. To test whether full-body tasks, which require considerable space, scientific equipment, and personnel, could be conducted more efficiently in a virtual reality (VR) setup, we faithfully reproduced the task in VR with a second group of participants. Results showed that participants walked straight through wider apertures but initiated body rotation below a critical width. The critical width shifted with order, suggesting an inverse hysteresis effect. This inverse hysteresis was confirmed in the second participant group in the virtual environment. However, VR participants rotated at wider apertures, indicating a more cautious behavior. Comparable behavioral patterns across environments support the feasibility of VR for full-body movement studies. Nevertheless, validating critical findings in a real environment seems advisable. The observed inverse hysteresis has only been described in perception research so far, where it indicates repetition suppression. Our findings suggest that repetition suppression may also apply to motor planning, especially when mechanical costs are high.

## 1 Introduction

Repetitive arm movements are a key element of various sports, such as tennis, golf, or darts. While the goal that an athlete wants to achieve with each movement might vary (e.g., switching from a short to a deep shot in tennis), our motor system has a tendency to persist in the previous movement kinematics, a phenomenon known as *motor hysteresis* (1). This persistence occurs even in simple reaching tasks, for example, when participants open a series of drawers. In a descending sequence, they start in a prone hand posture at the top drawer and then persist in a more prone posture. In an ascending sequence, they start and persist in a more supine hand posture. Due to this persistence, posture at the central drawers depends on the order, with more prone postures in descending sequences (2–4).

Such hysteresis effects in posture selection have been reproduced in a large number of studies using binary (1, 5, 6) and continuous (7–12) tasks. Hysteresis effects have also been found in limb selection, both for individual limb segments (13) and whole limbs (8, 14, 15). Weiss and Wark (16) had cotton-top tamarin monkeys reach through a hole for marshmallow pieces. When the pieces were presented in ordered progressions, the monkeys continued reaching with the initially selected limb at the central positions, thus exhibiting limb selection hysteresis.

Another series of reaching studies addressed hysteresis in reach path planning (17–21). Jax and Rosenbaum (18, 19) had participants perform a center-out pointing task while circumventing obstacles in some trials. In trials without an obstacle, hand paths remained more curved if an obstacle had recently been circumvented, indicating path planning hysteresis.

A key predictor for the occurrence of hysteresis appears to be the predictability of the sequences. While path curvature hysteresis in the study by Jax and Rosenbaum (18) was the same in predictable and unpredictable sequences of obstacles, path selection hysteresis in a study by Warburton et al. (21) was significantly reduced when switching from ordered to randomized sequences. Similar disruptive effects of unpredictable sequences have been found for posture selection hysteresis. Schütz et al. (3) had participants open drawers in randomized and ordered sequences. Hysteresis was only found in the ordered sequences. In two more recent studies, the authors applied a mathematical model to quantify the percentage of motor plan reuse, and found that it dropped from ∼20% in ordered sequences to ∼2% in randomized sequences (7, 10).

A common feature of all previously mentioned hysteresis studies is that they were limited to reaching movements. Indeed, only a few studies investigated hysteresis for other movements, focusing on coordination patterns of full-body movements. The majority of these studies investigated hysteresis for gait transitions: Hreljak (22) found a higher point-of-change (PoC) for the walk-run transition in increasing velocity sequences than for the run-walk transition in decreasing ones. However, later studies showed reverse (23, 24) or inconsistent hysteresis effects (25). Two other studies investigated phase transitions of coordination patterns in a rhythmic rope-skipping task (26) and a knee-bending task (27). Coordination patterns persisted when the movement frequency was systematically shifted, indicating full-body hysteresis.

To date, all studies demonstrating hysteresis in full-body movements investigated transitions between cyclic coordination patterns. These findings can be explained by dynamical systems theory (1, 28) which assumes that stable coordination patterns emerge as attractor states through self-organization processes within a network of interconnected biological systems. Hysteresis thus could, for example, result from low-pass properties of the muscles. However, muscle properties cannot be the sole origin of hysteresis: Van der Wel et al. (20) had participants touch targets on a table-top in serial progression and clear obstacle in random trials. After an obstacle, jump height only gradually returned to the baseline, even if participants cleared the obstacle with one hand and continued the progression with the other.

Therefore, several studies have proposed the cognitive planning system as an alternative cause of hysteresis. According to the *plan-modification hypothesis* (29), motor planning incurs cognitive costs. In a reaching movement, for instance, these costs may arise from the sensorimotor transformations required to convert the desired final posture into a muscle activation pattern (30). In repetitive tasks, however, planning costs can be reduced by retaining the prior movement plan for the next movement (5), resulting in a persistence of the prior state.

According to *Bayesian inference theory*, the brain constantly integrates incoming sensory data (the *likelihood* of the sensory evidence) with existing knowledge (the *prior*) to update its predictions (the *posterior*; cf. (31)). If transition probabilities between actions were updated in the same manner, movement selection would also be biased towards the *prior* state (32). Alternatively, state persistence could also result from *reinforcement learning*. If a reward were coupled to each movement, action selection in repetitive tasks would be biased towards previously rewarded movements (33). In a binary path selection task (circumventing an obstacle on the left or right), Warburton et al. (21) demonstrated that the measured persistence in the previous path could be replicated by a reinforcement learning model.

All of these cognitive approaches could potentially explain hysteresis in discrete (non-cyclic) posture selection tasks. Therefore, if hysteresis were a motor planning principle rooted in the cognitive system, it should not be limited to discrete reaching or cyclic full-body movements, but also apply to discrete full-body posture selection.

Therefore, a central aim of the current study was to test for hysteresis in a discrete full-body posture selection task. The lack of studies on hysteresis in full-body posture selection is surprising, as a large number of experimental paradigms are available. Regarding binary posture selection, van der Meer (34) asked participants to walk beneath a horizontal barrier and measured the height below which they would duck. For adults, the critical barrier-to-height (*B*/*H*) ratio was 1.04. Similar *B*/*H* ratios were found in subsequent studies (35, 36). Comalli et al. (37) asked participants to either go over or under a horizontal bar presented at different heights. For adults, the transition point between behaviors was at a bar-to-height ratio of approximately 0.43.

Regarding continuous posture selection, Warren and Whang (38) asked participants to pass through a vertical aperture while measuring shoulder rotation. The authors found that critical aperture width to initiate a shoulder rotation correlated with shoulder width, and they calculated a critical aperture-to-shoulder-width (*A*/*S*) ratio of 1.30. Below the critical threshold, shoulder rotation angle was inversely related to the *A*/*S* ratio. This inverse relationship was confirmed in two subsequent studies (39, 40). For the critical *A*/*S* ratio, Wilmut and Barnett (40) found a similar value of 1.30, whereas Franchak et al. (35) measured a considerably lower value of 1.10 in the same task. Almost all of the aforementioned studies used randomized sequences of conditions and did not test for hysteresis. Warren and Whang (38) used ordered sequences of *A*/*S* ratios, but did not test for “order” as a factor. Thus, this will be the first study to test for posture hysteresis in discrete full-body posture selection.

A major disadvantage of full-body posture selection tasks is the need for setups built to full-body scale, which involves extensive metal and wood constructions, considerable lab space, and a high personnel requirement to adjust the conditions between trials. These problems can be addressed by conducting the experiments in a virtual reality (VR) environment, using a modern VR system. These systems create a high sense of immersion (41) by measuring head kinematics in real time and by coupling the visual input to the participants' movements (42). They also provide a high degree of ecological validity through complex, naturalistic scenarios that can be created in modern game engines (42, 43), while still maintaining perfect control of the environment and allowing rapid changes of the experimental conditions between trials. Due to these advantages, VR systems are widely used in studies on cognition, gait and navigation (42, 44), rehabilitation (45), and sports.

In sports in particular, there are a number of studies that compared full-body movements between real and virtual environments. Often, response times and movements kinematics were similar in both environments (46, 47). However, participants in VR tended to move more slowly (48–50), exhibited greater motor variability (47, 49), and considered the task to be more difficult (48, 51). In a study on collision avoidance, Gérin-Lajoie et al. (52) measured the personal space, that is, the distance participants maintained from an obstacle, in both a real and virtual environment. The authors found that the overall shape was the same in both conditions, but that the size of the personal space was larger in VR.

A few studies also addressed the question in how far the visualization in the virtual environment affected the movement kinematics. Pastel et al. (53) tested participants in three different tasks: balancing, grasping, and throwing. Participants were provided with a full, partial, or no visualization of their own body. Visual feedback affected movement quality and completion time in the balancing and grasping task, but not in the throwing task. These findings indicated that the benefit of visual feedback was task-dependent. A full visualization of the body places greater demands on the available equipment (a full motion capture system is required in contrast to a VR headset only) and on the preparation of the participants (a motion capture suit and a full marker set are required). We therefore wanted to test whether visual feedback of the full body was necessary to replicate real world behavior in a posture selection task.

In the current study, we asked whether hysteresis was a general principle for cost reduction, that not only applies to reaching movements but to full-body movements as well. To this end, we created a full-body posture selection task with a continuous measure of posture: Participants passed through apertures of different widths while we measured their body rotation. Aperture widths were varied in randomized and ordered sequences. We expected an increasing body rotation below a critical aperture-to-width (*A*/*X*) ratio, resulting in a significant main effect of “*A*/*X* ratio” (38, 39). If hysteresis were present in such a continuous full-body posture selection task, we expected a significant main effect of the factor “order”, indicating a persistence in the previous posture. As previous studies have demonstrated that predictability is a prerequisite for hysteresis, we further expected an interaction between “condition” (randomized vs. ordered) and “order” and a greater hysteresis effect in the ordered sequences.

To test whether full-body planning tasks could be conducted more efficiently (less space, material, and personnel requirements) in VR, we faithfully replicated the setup in a virtual environment in a second experiment with a new group of participants. We expected similar kinematics in both environments (46, 47) but a more cautious behavior (48, 49) and a larger personal space (52), which could result in a higher critical *A*/*X* ratio in the virtual environment. This should be reflected by a significant main effect of “environment”. We further tested if movement kinematics in VR were affected by the body visualization by providing participants with a full or no visualization of their own body. If no significant differences were found for the main effect of “body visible” and its interactions, the more efficient (no real time motion capture, less preparation) feedback could be used in future studies.

## 2 Materials and methods

### 2.1 Power analysis

An *a priori* power analysis was calculated using the SPSS (28, IBM Corp., Armonk, NY) MANOVA procedure (54), which requires the number of participants for each group, means and standard deviations for the dependent variables, and correlations between the variables. Values were taken from the ordered pretest of the study by Schütz and Schack (2), who found hysteresis in a sequential drawer opening task. In their study, posture was measured as hand angle projected onto the drawer surface. Thus, the dependent variable was comparable to the projected shoulder, thorax, and pelvis angles used in the current study. Presuming a comparable, that is, large, effect size in the current study, 15 participants should be sufficient to achieve a power of over.95 for detecting a main effect of a within subject factor “order”, indicating hysteresis.

### 2.2 Participants

In Experiment 1 (real environment), 27 participants [15 female, 12 male, age 24.3 ± 2.5 (SD) years] were measured. Twenty-five participants were right handed [handedness score (HS): 0.98 ± 0.06] and two left handed (HS: −1.00 ± 0.00) according to the revised Edinburgh inventory (55). In Experiment 2 (virtual environment), 21 participants (12 female, 9 male, age 23.3 ± 3.0 years) were measured. Eighteen participants were right handed [handedness score (HS): 0.96 ± 0.12] and three left handed (HS: −1.00 ± 0.00). All participants were recruited at Bielefeld University and participated in exchange for either course credit or a participation fee (10 €). They reported no known neuromuscular disorders and were naïve to the purpose of the study. Before the experiment, each participant read a detailed description of the study, a data protection declaration, and provided written informed consent. The study was approved by the Bielefeld University ethics committee (proposal EUB-2022-200) and conformed to the 1964 Declaration of Helsinki (56).

### 2.3 Setup

**Figure 1:**
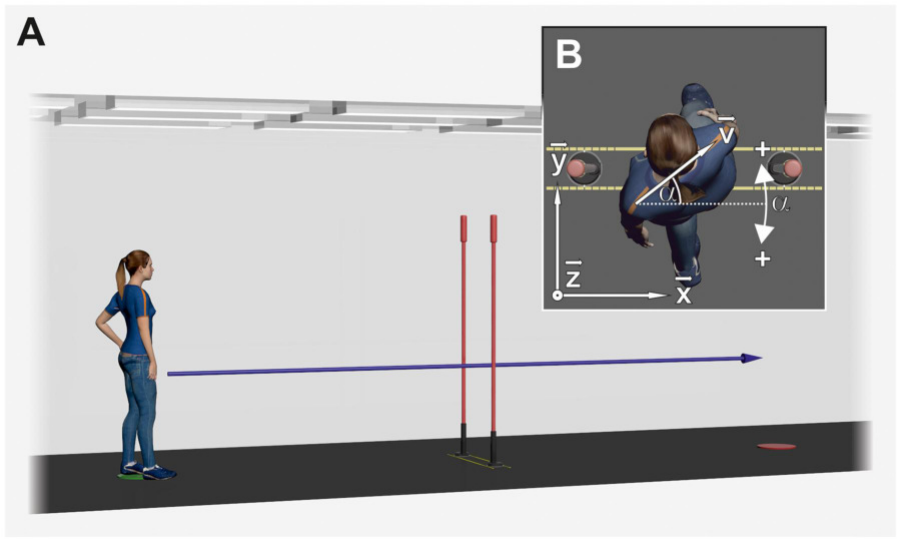
**(A)** schematic of the experimental setup. **(B)** Segment rotation angle *α*. The projection of the segment direction vector ***v*** onto the floor (***x***-***y***-plane) at the moment of aperture passing was used to calculate *α*.

In Experiment 1 (real environment), the setup consisted of two slalom poles (foot: 3 cm high, 12 cm diameter; pole: 180 cm high, 3 cm diameter) that were placed at the halfway point of a 5 m walkway (Figure 1A). Two blue rubber disks (30 cm diameter) on the floor marked the start and end position at each end of the walkway. The slalom poles created an aperture that could be varied in 5 cm steps from 35 cm (width 1) to 75 cm (width 9), thus creating nine different aperture widths through which participants had to pass.

The placement of the poles and the aperture width were controlled via two parallel strips of yellow adhesive tape that were attached 12 cm apart (Figure 1B). The slalom poles were placed between these strips and, for each pole, two white marks on either side of the foot had to be aligned with two black marks on the strips. For each pole, nine black marks on each side marked where the pole had to be positioned to create the correct aperture width.

In Experiment 2 (virtual environment), the setup was faithfully reproduced in the Unity game engine (2022.3.48f1, Unity Technologies, San Francisco, CA). Figure 1A depicts a render of the virtual scene used in the experiment. The virtual environment was presented with a Valve Index virtual reality headset (Valve Corporation, Bellevue, WA) at a resolution of 1,440 × 1,600 pixels with a 120 Hz refresh rate.

A rigid body with four retro reflective markers was attached to the headset and tracked with a Vicon Shogun motion capture system (v1.12.0, Vicon Motion Systems, Oxford, UK) with 16 Vicon Vero v2.2 cameras at a 100 Hz recording frequency. In the Shogun software, a rigid segment (or *prop*) was created for the headset, with its origin on the bridge of the nose and its ***x***-axis pointing right, ***y***-axis upward, and ***z***-axis backward. Motion data of the headset prop was then streamed to the Vicon client (part of the Vicon Unity plugin, v1.3.1) in Unity and processed by the *HMDScript* component to fuse the position data from Shogun with the inertial measurement data from the Valve Index. Thus, the position and orientation of the virtual environment were perfectly aligned with the real environment.

Four character models of different sizes were created for both females (150, 160, 170, 180 cm) and males (160, 170, 180, 190 cm) in MakeHuman (v1.3.0, the MakeHuman team) and attached to a custom skeleton rig in Blender (v4.1, Blender foundation, Amsterdam, Netherlands) that matched the skeleton rig (i.e., the bone names and bone hierarchy) of Vicon Shogun. In the Shogun software, a skeleton (or *subject*) was created for each participant, based on the default *FrontWaist3Fingers* marker set with 59 retroreflective markers. Motion data of the subject (position and orientation of each bone) were streamed to the Vicon client in Unity and processed by a custom-written C# script to map the motion data onto the character model. The custom-written script mapped both the orientations and the positions (i.e., lengths) of the bones and, thus, faithfully reproduced the body dimensions of the participant in the virtual environment. The script further added a rigid body and a capsule collider to each bone.

Rigid bodies and capsule colliders were also added to the virtual slalom poles to ensure that the participants could actually knock them over if they did not rotate their bodies sufficiently when passing through the aperture. The start and end position were updated to change their color from red to green when the participants (i.e., the headset) were directly above them, to provide additional visual feedback in the virtual environment.

### 2.4 Preparation

In both experiments, participants first read the study description and data protection declaration and provided written informed consent. Next, they changed into tight-fitting clothing and sneakers. Their shoulder width (between the left/right great tubercle) and hip width (between the left/right greater trochanter) were measured with a body caliper (WZ101, Uniprox GmbH, Zeulenroda, Germany).

In Experiment 1, ten retroreflective markers were attached to the thorax and pelvis of the participants: left and right *Articulatio acromioclaviculare* (L/R AC), *Processus spinosus* of the 7th cervical and 8th thoracic vertebra (C7 and T8), *Incisuria jugularis* (IJ), *Processus xiphoideus* (PX), left and right *Spina iliaca anterior superior* (L/R ASI) and *Spina iliaca posterior superior* (L/R PSI). A static calibration of the participants standing in an A-pose (palms down) was done in Vicon Nexus (v2.16.0, Vicon Motion Systems, Oxford, UK). Participants read the task instructions and could clarify open questions with the experimenter before they assumed the start position, facing away from the aperture. The preparation phase in Experiment 1 took approximately 20 min.

In Experiment 2, participants additionally put a motion capture suit over their tight-fitting clothing. Fifty-nine retroreflective markers were attached to the participants in accordance with the default *FrontWaist3Fingers* marker set of Vicon Shogun. A static A-pose-calibration and a range of motion of the participants was recorded in Vicon Shogun. The character model most closely matching the size and gender of the participants was activated in Unity. Participants read the task instructions and could clarify open questions with the experimenter. Next, the headset was adapted to the participants and they were given a 2 min familiarization phase in the virtual environment. During the familiarization phase, participants were encouraged to walk back and forth between the start and end position until walking in the virtual environment felt natural. Then, they assumed the start position, facing away from the aperture. The preparation phase in Experiment 2 took approximately 35 min.

### 2.5 Procedure

Experiment 1 consisted of 2 tasks. Each task contained 5 or 10 sequences of 9 trials each. A trial was defined as a single passing through the aperture. The participants started each trial on the start position, facing away from the aperture. The participants then had to 1) turn 180° on the start position, 2) walk to the end position at normal speed, starting with their right foot, 3) pass through the aperture without knocking over the slalom poles, and 4) stop at the end position, facing away from the aperture. Two research assistants then aligned the slalom poles with the correct marks for the next trial. The end position of the current trial then became the start position of the next trial. If participants knocked over a slalom pole during a sequence, the sequence was aborted and restarted.

In Task 1, participants performed 5 sequences of the 9 aperture widths [5 repetitions × 9 aperture widths = 45 trials]. In each sequence, the 9 aperture widths were presented in a pseudo-randomized order, that is, each width was presented once (e.g., 2,7,6,8,4,3,1,5,9). To this end, a list of 5 pseudo-random permutations [Mersenne twister algorithm (57)] was created before the experiment for each participant. Referring to this list, the research assistants updated the positions of the slalom poles once the participants had reached the end position.

In Task 2, participants performed 10 ordered sequences of the 9 aperture widths: 5 increasing (1,2,3,4,5,6,7,8,9) and 5 decreasing (9,8,7,6,5,4,3,2,1) sequences [2 orders × 5 repetitions × 9 aperture widths = 90 trials]. The sequence order was pseudo-randomized (see above) with no more than two repetitions of the same order in a row (e.g., d,i,d,i,i,d,i,i,d,d). Again, the research assistants updated the positions of the slalom poles after each trial in accordance to an individual sequence/trial list for each participant.

All participants conducted the randomized task (Task 1) first, to get accustomed to the experiment. Posture selection in randomized tasks is unaffected by hysteresis (3, 10) and, thus, should not affect posture in the subsequent ordered task (Task 2). Participants had a resting period of 30 s between each sequence and of 2 min after each set of 5 sequences.

Experiment 2 consisted of 4 tasks. Each task contained 4 or 8 sequences of 9 trials each. A single trial in Experiment 2 was identical to Experiment 1. After each trial, the positions of the slalom poles in the virtual environment were updated by the experimenter with a keypress. If participants knocked over a slalom pole during a sequence, the trial was automatically marked as failed and removed from the later data analysis. The order of the tasks and the sequences, the positions of the slalom poles, the start and end of each trial, and the marking of failed trials was controlled by the UXF toolbox (43).

In Task 1 (no visible body) and Task 3 (visible body), participants performed 4 sequences of the 9 aperture widths each [4 repetitions × 9 aperture widths = 36 trials per task]. In each sequence, the 9 aperture widths were presented in a pseudo-randomized order. To this end, 4 pseudo-random permutations of the 9 aperture widths were created before the experiment for each participant and stored as csv-files. Based on these files, UXF updated the positions of the slalom poles after each trial. In Task 2 (no visible body) and Task 4 (visible body), participants performed 8 ordered sequences of the 9 aperture widths each: 4 increasing and 4 decreasing sequences [2 orders×4 repetitions × 9 aperture widths = 72 trials per task]. Sequence and repetition rules were identical to Experiment 1, and sequence lists for each participant were stored as csv-files.

To test whether behavior in the virtual environment depended on the visibility of the own (virtual) body, we further varied the factor “body visible” in Experiment 2. In Tasks 1 and 2, the virtual body and the colliders were deactivated, thus replicating a simple VR setup with headset only. In Tasks 3 and 4, the virtual body and the colliders were activated, thus providing real-time visual feedback and physical interactions with the environment, which requires a considerably more expensive VR setup with real-time full-body motion capture. In these 2 tasks, participants could actually knock over the virtual slalom poles, similar to the real environment. The order of Tasks 1 and 2 and Tasks 3 and 4 was counterbalanced across participants; the order of the randomized and ordered condition was fixed (as in Experiment 1).

Participants had a resting period of 30 s between each sequence and of 2 min after each set of 4 sequences. During the 2 min breaks, participants were asked to remove the headset and rest their eyes.

The experimental phase took approximately the same time in the real (Experiment 1) and virtual (Experiment 2) environment, that is, 50 min. This was due to the fact that the number of trials was higher in Experiment 2, but the updating of the aperture widths between trials took less time.

### 2.6 Data processing

In Experiment 1, marker trajectories were reconstructed in Vicon Nexus v2.16.0, labeled manually, and exported to MATLAB (2021b, The MathWorks, Natick, MA) for data analysis. In Experiment 2, reconstructed and prelabeled marker trajectories in Vicon Shogun v1.12.0 could be directly exported to MATLAB. Marker names of the *FrontWaist3Fingers* marker set were adapted to the marker names used in Experiment 1, to use the same subsequent computations. The laboratory's coordinate system in both experiments was defined with the ***x***-axis pointing to the right, the ***y***-axis pointing to the front, and the ***z***-axis pointing upwards while standing on the start position facing the aperture (Figure 1B).

To identify the moment when participants were passing through the aperture, we first calculated the segment center trajectories of the shoulder girdle (SC), thorax (TC), and pelvis (PC; Table 1) To identify the moment of aperture passing for each segment, the distance in the ***y***-direction (i.e., the walking direction; Figure 1B) between the aperture center and the segment center was analyzed. The distance graph exhibited 9 local minima for each sequence. The corresponding frames of these minima were used to calculate the segment rotation angles *α*.

**Table 1:** Position and direction vectors used for the rotation angle calculation.

Description	Abbreviation	Computation
Positions		
Shoulder center	SC	(LAC + RAC)/2
Thorax center	TC	(PX + IJ + C7 + T8)/4
Pelvis center	PC	(LASI + RASI + LPSI + RPSI)/4
Direction vectors		
Global *z*-axis	***z***	
Shoulder direction vector	***v***_**shoulder**_	RAC − LAC
Thorax support vector	***s***_**thorax**_	(PX + IJ)/2 − (C7 + T8)/2
Thorax direction vector	***v***_**thorax**_	***s***_**thorax**_ × ***z***
Pelvis support vector	***s***_**pelvis**_	(LASI + RASI)/2 − (LPSI + RPSI)/2
Pelvis direction vector	***v***_**pelvis**_	***s***_**pelvis**_ × ***z***

To calculate the rotation angle *α*, a segment direction vector ***v*** was defined for each segment. For the shoulder girdle, ***v*** was defined as the connection vector from the left to the right acromion (Table 1), thus pointing along the ***x***-axis if participants passed the gap without upper body rotation (Figure 1B). To define comparable direction vectors for the thorax and pelvis, we first defined support vectors ***s*** that pointed forward and then calculated the cross product of ***s*** with the global ***z***-axis (Table 1).

From the vector components v_x_ and v_y_, the segment rotation angle *α* was calculated with the four-quadrant inverse tangent function of MATLAB. This was equivalent to a projection of the segment direction vector onto the floor (***x***-***y***-plane, Figure 1B). The absolute value of *α* was used as the dependent variable.

The segment rotation angle *α* was zero when participants passed straight through the aperture and increased if the segment was rotated to the left or right. Participants tended to rotate their body away from the leading foot when stepping through the aperture. Whether they rotated to the left or right was largely determined by the participants’ step lengths. Within each participant, rotation direction was consistent, as we asked them to always start the movement with their right foot. To render the data of left- and right-rotating participants comparable, the absolute value of *α* was used.

Data were aggregated over participant, task, and experiment. In Experiment 1, 4 of 27 participants knocked over a slalom pole and, thus, had to abort and restart one sequence. Trials from aborted sequences (1.0% of all trials) were excluded from the analysis. Similarly, in Experiment 2, trials that were marked as failed (6.5%) were removed. Therefore, only successful passes were included in the analysis of both experiments. To render the data more comparable across participants of different body dimensions, the aperture width was divided by the shoulder width of each participant (*A*/*S* ratio) for the analysis of the shoulder and thorax rotation, and by the hip width (*A*/*H* ratio) for the pelvis rotation. Thus, participants of different shoulder widths should initiate their shoulder rotation at a similar *A*/*S* ratio. The same applies to thorax and pelvis rotation. *A*/*S* ratio and *A*/*H* ratio (from now on termed *A*/*X* ratio for simplicity) were then centered around the mean to be used as fixed effects in the models.

Five additional independent variables were used as fixed effects in the models: “environment” (real or virtual), “condition” (randomized or ordered), “order” (increasing or decreasing *A*/*X* ratio), “fatigue” (number of sequences carried out since the start of the experiment), and “body visible” (false or true, only defined in the virtual environment). These variables were scaled to values between −1 and +1.

In the randomized conditions, “order” was determined based on the *A*/*X* ratio of trial *n* − 1: if the *A*/*X* ratio of trial *n* − 1 was lower, “order” of trial *n* was set to increasing; if it was higher, “order” was set to decreasing. Thus, “order” in the randomized conditions was comparable to “order” in the ordered conditions. As no trial *n* − 1 was available for the first trial of each sequence, these trials were removed from the data sets of both the randomized and the ordered conditions.

Segment rotation angles were analyzed with a generalized linear mixed-effects model (GLMM) with a logit link function. The logit link function was used to better capture the clear bend in the rotation angle data found in an aperture task (38), resulting from a straight posture for larger *A*/*X* ratios and an increasingly rotated posture below a critical *A*/*X* ratio. To make the dependent variables (shoulder, thorax, and pelvis angle) compatible to the logit link function, the rotation angles for all participants were divided by their respective maximum rotation angle. Thus, the dependent variables were scaled between 0 (no rotation) and 1 (maximum rotation).

### 2.7 Statistical analyses

To test (1) if participants persisted in their previous postures and (2) if participants' postures differed in the real and virtual environment, we calculated GLMMs (logit link function) on the scaled rotation angles of shoulder, thorax, and pelvis, with “*A*/*X* ratio”, “environment” (real or virtual), “condition” (randomized or ordered), “order” (increasing or decreasing), and “fatigue” as fixed effects, and “participant ID” as a random effect. We added two interactions to the model: one between “environment” and “order” to test whether a potential hysteresis effect differed between the real and virtual environment, and one between “condition” and “order” to account for the fact that hysteresis effects have been found to be significantly smaller in randomized than in ordered sequences of trials (10, 21). For this model, all trials from Experiment 1 (real environment) and only trials with a visible body and active colliders from Experiment 2 (virtual environment) were used, based on the assumption that behavior in VR would be most similar to real-world behavior with real-time visual feedback of the own body and physical interactions with the environment.

To test (3) whether real-time visual feedback of the own body and physical interactions with the virtual environment were necessary to reproduce real-world behavior or if simple head-tracking was sufficient, we calculated GLMMs on the scaled rotation angles of shoulder, thorax, and pelvis, with “*A*/*X* ratio”, “body visible” (false or true), “condition” (randomized or ordered), “order” (increasing or decreasing), and “fatigue” as fixed effects, and “participant ID” as a random effect. Two interactions were added to the model: one between “body visible” and “order” to test whether a potential hysteresis effect differed depending on the visual feedback, and one between “condition” and “order” to account for potential differences in hysteresis effects between randomized and ordered sequences of trials. For this model, only the trials from Experiment 2 were used.

As a reviewer suggested, the posture chosen by the participants may be influenced not only by the previous postures or the perceptual judgement of aperture width, but also by intrinsic variance in movement execution (44, 47, 49) that could affect task success (33). Therefore, to test (4) whether posture variance may have influenced participant behavior in our task, we calculated the standard deviation (SD) as a measure of variance. For the first dataset (comparison of real and virtual environment), we calculated the SDs of the scaled rotation angles of shoulder, thorax, and pelvis for each unique combination of “*A*/*X* ratio”, “environment”, “condition”, “order”, and “participant ID”. In order to have multiple repetitions of each combination available for the SD calculation, the factor “fatigue” had to be aggregated. We then calculated a linear mixed-effects model (LMM) that was identical to the GLMM used for the posture analysis, except for the fixed effect of “fatigue”. As a linear function for “*A*/*X* ratio” did not match the variance pattern well, we added a logit derivative function for “*A*/*X* ratio” to the model to align it with the logit function used for the posture. For the second dataset (effect of visual feedback), we calculated the SDs for each unique combination of “*A*/*X* ratio”, “body visible”, “condition”, “order”, and “participant ID” and then again calculated an LMM identical to the GLMM used for the posture analysis (with a logit derivative function for “*A*/*X* ratio” and without a fixed effect of “fatigue”).

The LMMs and GLMMs were calculated in R (v4.3.2; R Core Team, 2023) with the lme4 package (58). Effect sizes for the LMMs were calculated with the r2glmm package (59), using the method of Nakagawa and Schielzeth (60). Effect sizes for the GLMMs were calculated as odds ratios.

## 3 Results

### 3.1 Comparison of the real and virtual environment

**Figure 2:**
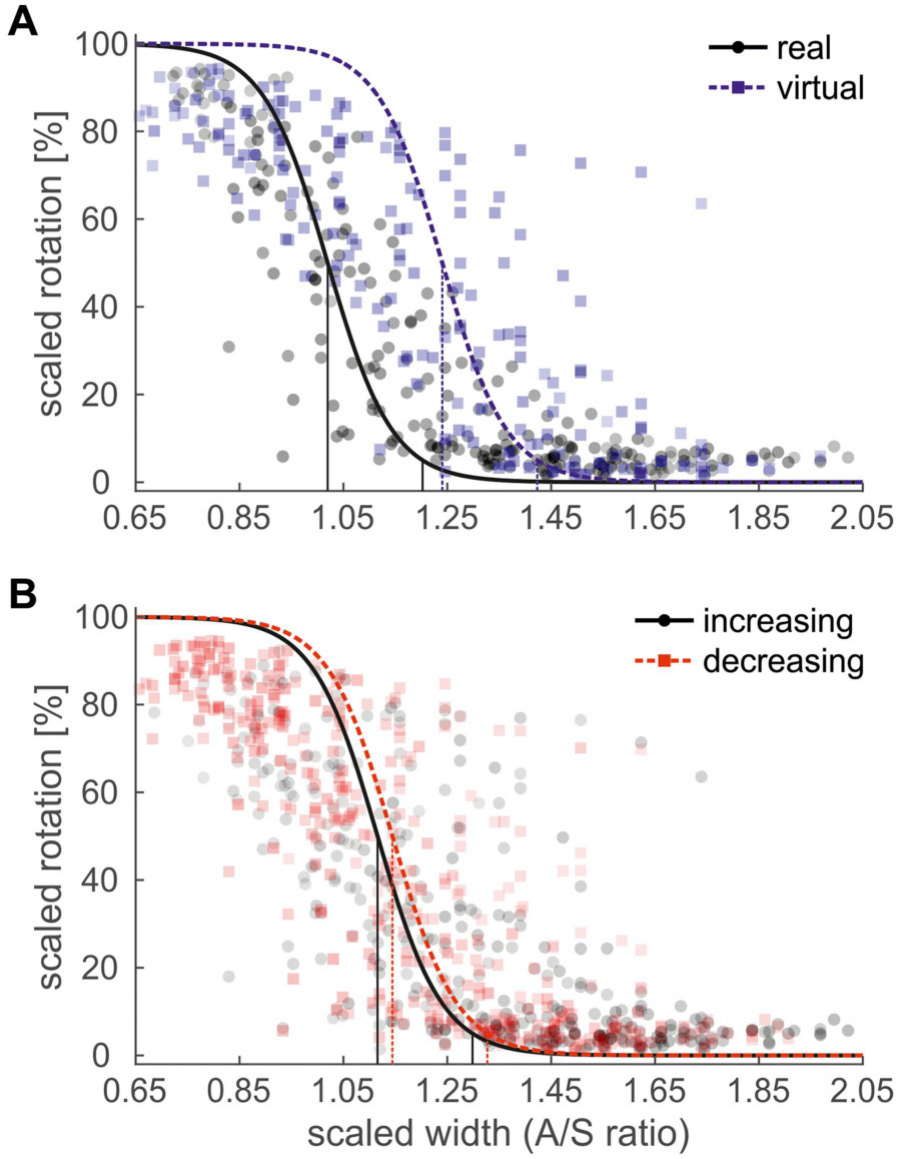
Scaled shoulder rotation, plotted against “*A*/*S* ratio”, **(A)** split by “environment” **(B)** split by “order”. *Markers* show the average rotation values of a participant at each of their 9 individual *A*/*S* ratios. *Thick lines* show the logistic curves of the fixed effects of the GLMM. *Thin, vertical lines* mark the points-of-change (50% rotation) and critical values (5% rotation). Note: *# of data points included in each marker is reflected by its face opacity, e.g., in panel **(A)**, up to 15 data points (corresponding to the 15 sequences) were averaged per participant and A/S ratio in the real environment, and up to 12 data points in the virtual environment.*

For the shoulder rotation, the GLMM comparing the real and virtual environment showed a significant main effect of “*A*/*X* ratio” (Table 2). Participants passed straight through the aperture for the higher *A*/*X* ratios. Below a critical *A*/*X* ratio, they had to use an increasingly rotated posture to pass through the aperture (Figure 2A). There were significant main effects of “environment” and “order”, and no interaction between the two (Table 2). Thus, both main effects could be interpreted. The point-of-change (i.e., 50% of the individual maximum rotation) in the real environment was at a significantly lower *A*/*X* ratio (102.4% of shoulder width) than in the virtual environment (124.5%; Figure 2A).

**Table 2:** GLMM results for the posture. Comparison between the real and virtual environment.

Fixed effect	Estimate	*Z*	*p*	Odds ratio
Shoulder				
***A*/*S* ratio**	−**16**.**111**	−**29**.**076**	**<.001**	**1.008 × 10** ^−**7**^
**environment**	**+1**.**777**	**+6**.**064**	**<.001**	**5**.**913**
condition	−0.051	−0.511	.610	0.950
**order**	**+0**.**230**	**+3**.**543**	**<.001**	**1**.**258**
fatigue	+0.209	+0.797	.425	1.232
environment:order	−0.087	−1.500	.134	0.916
Condition:order	−0.022	−0.344	.731	0.978
Thorax				
***A*/*S* ratio**	−**16**.**415**	−**28**.**791**	**<.001**	**7.430 × 10** ^−**8**^
**environment**	**+1**.**890**	**+6**.**652**	**<.001**	**6**.**620**
condition	−0.107	−1.061	.289	0.898
**order**	**+0**.**260**	**+3**.**966**	**<.001**	**1**.**298**
fatigue	+0.258	+0.981	.326	1.295
environment:order	−0.060	−1.013	.311	0.942
condition:order	−0.049	−0.765	.444	0.952
Pelvis				
***A*/*H* ratio**	−**12**.**213**	−**27**.**898**	**<.001**	**4.965 × 10** ^−**6**^
**environment**	**+2**.**020**	**+6**.**578**	**<.001**	**7**.**537**
condition	−0.006	−0.060	.952	0.994
**order**	**+0**.**257**	**+3**.**792**	**<.001**	**1**.**293**
fatigue	−0.122	−0.455	.649	0.885
environment:order	−0.048	−0.801	.423	0.954
condition:order	−0.053	−0.788	.430	0.949

Significant differences are printed in bold.

**Figure 3:**
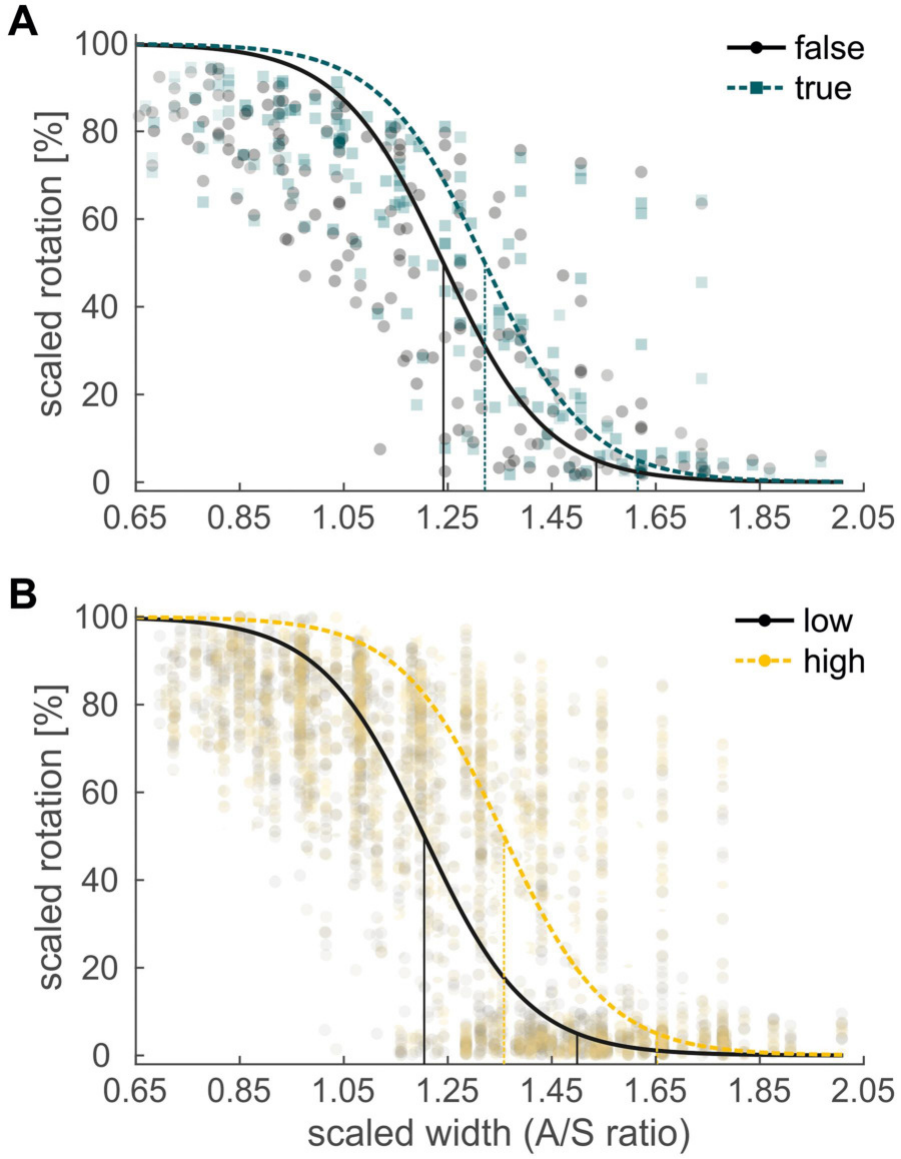
Scaled shoulder rotation, plotted against “*A*/*S* ratio”, **(A)** split by “body visible” **(B)** split by “fatigue”. *Markers* show the average rotation values of the participants at each of their 9 individual *A*/*S* ratios. *Thick lines* show the logistic curves of the fixed effects of the GLMM. *Thin, vertical lines* mark the points-of-change (50% rotation) and critical values (5% rotation). Note: *# of data points included in each marker is reflected by its face opacity, e.g., in panel **(A)**, up to 12 data points (corresponding to 12 sequences each) were averaged per participant and A/S ratio for the body invisible and the body visible condition. Face opacity in panel **(B)** was increased by a factor of 2 (in comparison to the other panels) to render the data points more visible.*

Since it is customary in these types of studies to report not the point-of-change (50% rotation), but the critical value below which a rotation is initiated (5% rotation), only critical values are reported for all subsequent results. However, both measures are depicted in the figures (Figures 2,3) and, due to the negligible interaction terms in the models (resulting in parallel curves), the offset is constant across the whole range of *A*/*X* ratios. The main effect of “environment” thus also indicated that the critical value in the real environment (120.7%) was significantly lower than in the virtual one (142.8%). Participants in the virtual environment initiated their shoulder rotation at a larger aperture width.

The main effect of “order” indicated that the critical value in increasing sequences of *A*/*X* ratios was significantly lower (130.3%) than in decreasing sequences (133.2%; Figure 2B) of trials. Participants switched from a rotated to a straight posture at a smaller aperture width in increasing sequences, and from a straight to a rotated posture at a larger aperture width in decreasing sequences. Thus, participants did not persist in their previous posture (which would indicate hysteresis) but instead adopted their next posture earlier. The non-significant interaction between “environment” and “order” (Table 2; Supplementary Figures S1A,B) suggested that this inverse hysteresis effect did not differ between the real and the virtual environment, that is, in two independent participant groups. Presuming sufficient power, as indicated by the *a priori* power analysis, it can be concluded that behavior was similar in both environments. The non-significant interaction between “condition” and “order” (Table 2; Supplementary Figures S1G,H) indicated that no difference in the size of the inverse hysteresis effect was found between the randomized and ordered condition. In the randomized condition, “order” of each trial *n* was based on the *A*/*X* ratio of trial *n* − 1 (if the *A*/*X* ratio of trial *n* − 1 was lower, “order” was set to increasing; if it was higher, “order” was set to decreasing), and was thus comparable to the encoding in the ordered condition.

We did not find significant main effects of “condition” or “fatigue” (Table 2; Supplementary Figures S1C–F), suggesting that the critical values (i.e., the behavior of the participants) did not differ between the randomized and the ordered condition and also did not change over the course of the experiment. A full visualization of all the main effects and interactions in the model can be found in the Supplementary Figure S1.

All effect sizes were “very small”, except for the main effects of “*A*/*X* ratio” and “environment”, which were “large” (61). For the thorax and pelvis rotation, the GLMMs comparing the real and virtual environment qualitatively reproduced the results for the shoulder rotation, with significant main effects of “*A*/*X* ratio”, “environment”, and “order” and same signs for the corresponding estimates (Table 2). Thus, all significant shifts of the critical values were comparable to the reported results for the shoulder rotation.

### 3.2 Effects of visual feedback in the virtual environment

For the shoulder rotation, the GLMM comparing the effects of different visual feedback in the virtual environment showed a significant main effect of “*A*/*X* ratio” (Table 3). Participants used a straight posture for the higher and a rotated posture for the lower *A*/*X* ratios (Figure 3A). There were significant main effects of “body visible” and “order”, and no interaction between the two (Table 3). Thus, both main effects could be interpreted. The main effect of “body visible” indicated that the critical value without visual feedback of the own body (154.0%) was significantly lower than with visual feedback (162.0%; Figure 3A). Participants with feedback initiated their shoulder rotation at a larger aperture width.

**Table 3:** GLMM results for the posture. Effects of visual feedback in the virtual environment.

Fixed effect	Estimate	*Z*	*p*	Odds ratio
Shoulder				
***A*/*S* ratio**	−**10.014**	−**29.013**	**<.001**	**4.475 × 10** ^−**5**^
**body visible**	**+0** **.** **400**	**+7** **.** **629**	**<.001**	**1** **.** **490**
condition	+0.012	+0.192	.847	1.012
**order**	**+0** **.** **143**	**+2** **.** **507**	**.** **012**	**1** **.** **154**
**fatigue**	**+0** **.** **768**	**+8** **.** **110**	**<.001**	**2** **.** **156**
body visible:order	−0.041	−0.806	.420	0.960
condition:order	−0.067	−1.193	.233	0.935
Thorax				
***A*/*S* ratio**	−**10.321**	−**28.939**	**<.001**	**3.293 × 10** ^−**5**^
**body visible**	**+0** **.** **405**	**+7** **.** **640**	**<.001**	**1** **.** **499**
condition	−0.002	−0.036	.971	0.998
**order**	**+0** **.** **154**	**+2** **.** **662**	**.** **008**	**1** **.** **167**
**fatigue**	**+0** **.** **773**	**+8** **.** **062**	**<.001**	**2** **.** **166**
body visible:order	−0.065	−1.256	.209	0.937
condition:order	−0.081	−1.417	.156	0.922
Pelvis				
***A*/*H* ratio**	−**8.779**	−**29.224**	**<.001**	**1.540 × 10** ^−**4**^
**body visible**	**+0** **.** **502**	**+9** **.** **053**	**<.001**	**1** **.** **653**
condition	−0.088	−1.394	.163	0.916
**order**	**+0** **.** **141**	**+2** **.** **377**	**.** **018**	**1** **.** **152**
**fatigue**	**+0** **.** **743**	**+7** **.** **502**	**<.001**	**2** **.** **102**
body visible:order	−0.061	−1.147	.251	0.941
condition:order	−0.078	−1.323	.186	0.925

Significant differences are printed in bold.

The main effect of “order” (Table 3) indicated that the critical value in increasing sequences of *A*/*X* ratios was significantly lower (156.6%) than in decreasing sequences (159.4%). In the virtual environment, participants in increasing sequences switched postures at a smaller aperture width than in decreasing sequences, demonstrating an inverse hysteresis effect. There was no interaction between “body visible” and “order”, or between “condition” and “order” (Table 3), indicating that the size of the inverse hysteresis effect did not differ significantly depending on the visual feedback, or between the randomized and ordered condition. There also was no main effect of “condition”, indicating that the critical values did not differ significantly between the randomized and the ordered condition.

We found a significant main effect for “fatigue” (Table 3) in the virtual environment. Participants adjusted their behavior over the course of the experiment: The critical value to initiate a shoulder rotation significantly increased from the first (150.3%) to the last (165.7%; Figure 3B) sequence, that is, by 0.67% per sequence. With increasing fatigue, participants initiated their shoulder rotation at larger aperture widths.

Effect sizes were “very small”, except for the main effect of “*A*/*X* ratio”, which was “large”, and for the main effects of “body visible” and “fatigue”, which were “small” (61). For the thorax and pelvis rotation, again, the GLMMs comparing the effects of different visual feedback in the virtual environment reproduced the results for the shoulder rotation, with significant main effects of “*A*/*X* ratio”, “body visible”, “order”, and “fatigue” and same signs for the corresponding estimates (Table 3). Thus, all significant shifts of the critical values were comparable to the reported results for the shoulder rotation.

### 3.3 Analysis of the posture variance

**Figure 4:**
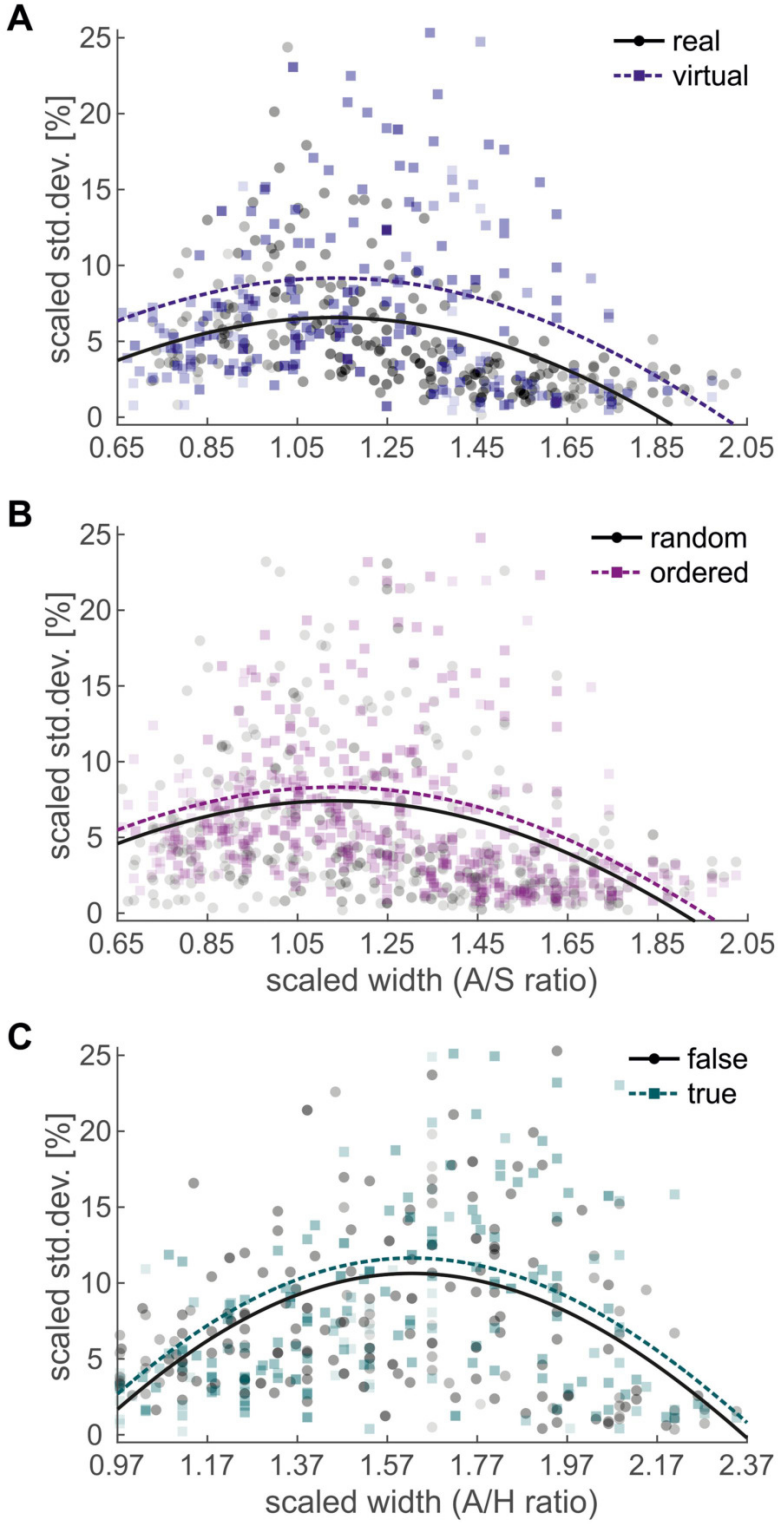
**(A,B)** Scaled shoulder standard deviation (SD), plotted against “*A*/*S* ratio”, **(A)** split by “environment” **(B)** split by “condition”. **(C)** *Scaled pelvis SD*, *plotted against* “*A*/*H* ratio”, split by “body visible”. *Markers* show the average SD of a participant at each of their 9 individual *A*/*X* ratios. *Thick lines* show the regressions of the fixed effects of the LMM.

For the shoulder SDs, an LMM with only a linear function for “*A*/*X* ratio” did not accurately reflect the pattern of variance, as variance was higher at the central *A*/*X* ratios and lower at the borders (Figure 4A). We therefore added a logit derivative function for “*A*/*X* ratio” to the variance model, to align it with the logit function used for the posture analysis. The expanded model achieved a considerably better fit (BIC: −3,286) than the linear model (BIC: −3,241) when comparing the real and virtual environment. The linear effect of “*A*/*X* ratio” was significant (Table 4) with a negative slope (−3.0% per *A*/*X* ratio). This indicated that SDs decreased as *A*/*X* ratios increased (Figure 4A). The logit derivative effect of “*A*/*X* ratio” was also significant (Table 4), demonstrating that SDs were higher for central *A*/*X* ratios, that is, where the shift between the rotated and the straight posture occurred (cf. Figure 2A).

**Table 4:** LMM results for the posture variance. Comparison between the real and virtual environment.

Fixed effect	Estimate	*t*	*p*	*R* ^2^ _β__*_
Shoulder				
***A*/*S* ratio linear**	−**0.030**	−**4.413**	**<.001**	**.** **016**
***A*/*S* ratio log.deriv.**	**+2** **.** **109**	**+7** **.** **367**	**<.001**	**.** **041**
**environment**	**+0** **.** **013**	**+4** **.** **911**	**<.001**	**.** **039**
**condition**	**+0** **.** **005**	**+2** **.** **549**	**.** **011**	**.** **005**
order	+0.001	+0.623	.534	<.001
environment:order	−0.002	−0.971	.332	.001
condition:order	<+0.001	+0.120	.905	<.001
Thorax				
***A*/*S* ratio linear**	−**0.031**	−**4.502**	**<.001**	**.** **017**
***A*/*S* ratio log.deriv.**	**+2** **.** **084**	**+7** **.** **304**	**<.001**	**.** **040**
**environment**	**+0** **.** **013**	**+5** **.** **235**	**<.001**	**.** **039**
**condition**	**+0** **.** **004**	**+2** **.** **332**	**.** **020**	**.** **004**
order	+0.001	+0.639	.523	<.001
environment:order	−0.001	−0.844	.399	.001
condition:order	<+0.001	+0.234	.815	<.001
Pelvis				
***A*/*H* ratio linear**	−**0.034**	−**6.850**	**<.001**	**.** **038**
***A*/*H* ratio log.deriv.**	**+0** **.** **868**	**+5** **.** **078**	**<.001**	**.** **020**
**environment**	**+0** **.** **012**	**+5** **.** **031**	**<.001**	**.** **039**
**condition**	**+0** **.** **003**	**+1** **.** **905**	**.** **057**	**.** **003**
order	+0.001	+0.712	.477	<.001
environment:order	−0.002	−1.107	.268	.001
condition:order	>−0.001	−0.176	.860	<.001

Significant differences are printed in bold.

We further found a significant main effect of “environment” (Table 4), indicating that the posture variance was lower in the real environment (6.4%) than in the virtual environment (9.0%; Figure 4A). A significant main effect of “condition” (Table 4) further indicated that the mean SD was lower in the randomized condition (7.2%) than in the ordered condition (8.1%; Figure 4B). All remaining fixed effects and interactions were not significant (Table 4), indicating that no differences in posture variance were found between increasing and decreasing orders. While the effect size for “environment” was “large” (61), the effect size for “condition” was “very small”. For the thorax and pelvis SDs, the LMMs comparing the real and virtual environment qualitatively reproduced the results for the shoulder SDs. However, for the pelvis, the main effect of “condition” was only close to significance (Table 4). Again, the models expanded by a logit derivative function performed better than the linear models.

When comparing the effects of visual feedback for the shoulder SDs, again, the expanded model achieved a considerably better fit (BIC: −2,100) than the linear model (BIC: −2,007). Both the linear and the logit derivative effect of “*A*/*X* ratio” were significant (Table 5). There was a significant interaction between “body visible” and “order” (Table 5), with a “very small” effect size (61). In the increasing sequences, posture variance was higher with visual feedback (11.6%) than without visual feedback (9.9%). In the decreasing sequences, posture variance was more similar (10.7 vs. 11.1%). For thorax and pelvis SDs, the expanded logit derivative models performed better as well. The LMM for the thorax SDs qualitatively reproduced the results for the shoulder SDs (Table 5). The LMM for the pelvis SDs, in contrast, showed no significant interaction of “body visible” and “order”, but a significant main effect of “body visible” (Table 5), with a “very small” effect size (61). Posture variance was higher with visual feedback (11.6%) than without (10.6%; Figure 4C).

**Table 5:** LMM results for the posture variance. Effects of visual feedback in the virtual environment.

Fixed effect	Estimate	*t*	*p*	*R* ^2^ _β__*_
Shoulder				
***A*/*S* ratio linear**	**+0** **.** **045**	**+4** **.** **184**	**<.001**	**.** **018**
***A*/*S* ratio log.deriv.**	**+4** **.** **853**	**+10** **.** **186**	**<.001**	**.** **090**
body visible	+0.003	+1.238	.216	.001
condition	+0.004	+1.417	.157	.002
order	+0.001	+0.322	.748	<.001
**body visible:order**	−**0.005**	−**2.077**	**.** **038**	**.** **004**
condition:order	+0.001	+0.512	.603	<.001
Thorax				
***A*/*S* ratio linear**	**+0** **.** **046**	**+4** **.** **242**	**<.001**	**.** **019**
***A*/*S* ratio log.deriv.**	**+4** **.** **995**	**+10** **.** **388**	**<.001**	**.** **093**
body visible	+0.004	+1.427	.154	.002
condition	+0.004	+1.463	.144	.002
order	+0.001	+0.393	.695	<.001
**body visible:order**	−**0.005**	−**2.125**	**.** **034**	**.** **004**
condition:order	+0.002	+0.600	.549	<.001
Pelvis				
***A*/*H* ratio linear**	**+0** **.** **024**	**+2** **.** **973**	**.** **003**	**.** **009**
***A*/*H* ratio log.deriv.**	**+3** **.** **515**	**+10** **.** **943**	**<.001**	**.** **103**
body visible	+0.005	+2.056	.040	.004
condition	+0.004	+1.425	.155	.002
order	+0.002	+0.874	.382	.001
**body visible:order**	−**0.005**	−**1.952**	**.** **051**	**.** **004**
condition:order	>−0.001	−0.162	.871	<.001

Significant differences are printed in bold.

## 4 Discussion

In the current study, we asked whether hysteresis was a motor planning principle rooted in the cognitive system and, thus, would apply to full-body posture planning. To this end, we tested participants in a continuous full-body posture selection task—passing through apertures of different widths—in both a real and virtual environment. If full-body posture selection were subject to hysteresis, we expected a significant main effect of “order”. By comparing the behavior in the real and virtual environment and with and without body visualization, we tested whether full-body planning tasks could be conducted more efficiently in VR without reducing scientific validity. We expected comparable movement kinematics, a more cautious behavior in the virtual environment, and potential differences in behavior depending on the visual feedback.

Results showed that participants walked straight through wider apertures but initiated a body rotation below a critical width (main effect of “*A*/*X* ratio”). This critical width shifted with order (main effect of “order”). However, the direction of the effect did not indicate a persistence of the previous posture but rather an inverse hysteresis effect. This inverse hysteresis was confirmed in a second participant group in the virtual environment (no interaction of “environment” and “order”). However, VR participants rotated at wider apertures (main effect of “environment”), indicating more cautious behavior in VR. Less cautious behavior was observed without visual feedback (main effect “body visible”), likely due to a lack of error feedback.

The full body-posture selection task used in the current study was introduced by Warren and Whang (38). The authors found a critical aperture-to-shoulder-width (*A*/*S*) ratio, below which participants rotated their upper body when passing through the aperture. Below the critical threshold, the shoulder rotation angle was inversely related to the *A*/*S* ratio (38–40). This pattern of results was reproduced in our study, resulting in a main effect of “*A*/*X* ratio” in the logistic model, which captured both the clear bend in the data and the inverse relationship below the critical threshold (Figure 2A). The pattern was found both in the real and virtual environment. The critical threshold in our real environment was 1.2, which matches the range reported for normal gait (1.1–1.3) in previous studies (35, 38, 40).

One interesting effect that has not been shown in previous studies, but which can be inferred from common sense, is that the inverse relationship between rotation angle and the *A*/*X* ratio cannot be maintained for very low *A*/*X* ratios. Instead, the rotation angles converge towards a maximum value. At this point, participants pass through the aperture sideways and do not increase their rotation any further. This convergence towards a stable maximum value was captured well by the logistic model in the current study (Figure 2A). The convergence presumably was not evident in the data of Warren and Whang (38), as the authors did not report their findings for the smallest aperture widths. In our study, it became even more evident in the virtual environment, where rotation angles were generally higher and, therefore, reached the point of convergence earlier (cf. Figure 2A; Figure 3).

The central question of the current study was whether hysteresis was present in a full-body posture selection task. To date, hysteresis in full-body tasks was only demonstrated for cyclic coordination patterns (22, 26, 27). For cyclic movements, hysteresis could be an emergent effect of the low-pass properties of the muscles (1, 28). There are, however, hysteresis effects which cannot be attributed to muscle properties (20) and, therefore, have been attributed to the cognitive planning system (21, 29, 32, 33). If hysteresis were indeed a planning principle rooted in the cognitive system, it should also apply to discrete posture selection in a full-body task.

To address this question, we had participants perform sequences with increasing and decreasing aperture widths, which should result in posture selection hysteresis. We found a significant main effect of “order”, making this the first study to demonstrate sequential effects in a non-cyclic full-body task (Figure 2B). However, the direction of the effect did not indicate a persistence in the previous posture but an inverse effect, which to date has only been demonstrated in one specific reaching task with children in a small subset of participants (62, 63). Presuming sufficient power (as indicated by the *a priori* power analysis), the non-significant interaction of “environment” × “order” (Table 2) in our study indicated that this inverse hysteresis effect was reproduced in direction and size in the real and in the virtual environment, that is, in two independent participant groups. Thus, it should not be considered an accidental finding.

A possible explanation for the inverse effect might be derived from perception research: Fischer and Whitney (64) found that, when participants had to judge the orientation of a sine grating, their orientation percept was biased towards the previous orientation. This indicated that, just like motor planning, perception was affected by hysteresis. Perceptual hysteresis has been found in a number of studies in the visual (65) and auditory domain (66, 67). In perception research, in contrast to motor planning, a second effect has been well investigated: repetition suppression (RS), which may be considered inverse hysteresis.

While hysteresis leads to a persistence of the previous percept and consequently to an increase in perceptual error, RS amplifies perceptual differences and reduces the perceptual error (31). RS results from a reduced firing rate of neurons in the temporal cortex (68) after repeated presentation of a stimulus, due to a long-lasting reduction in excitability (69). Fritsche et al. (70) demonstrated that both hysteresis and RS can co-occur in the same task: The percept in an orientation judgment task was repelled from the previous one (indicating RS), while the decision was biased towards the previous one (indicating hysteresis). Assuming that perception and motor planning share hysteresis as a common mechanism, the inverse hysteresis effect observed in the current study may suggest that this also applies to RS.

In an alternative theory proposed by Trapp et al. (31), hysteresis and RS are considered to be two sides of the same coin, with the recent past exerting different perceptual or motor effects depending on the context. Schütz and Schack (2), for example, varied the mechanical cost in a sequential grasp posture selection task and found that hysteresis was significantly reduced as mechanical cost increased. In their *cost-optimization theory* (11), the authors suggested that hysteresis reduces the cognitive cost of planning but increases the mechanical cost of execution. The amount of hysteresis that minimizes the total movement cost therefore depends on the ratio of both cost factors. In their theory, hysteresis was a modelled as a fixed percentage of reuse (PoR) of the previous motor plan. An increase in mechanical cost reduced the PoR.

While the authors only assumed that the PoR would converge to zero for high mechanical costs, our results suggest that even a shift to a negative PoR is possible, which would result in the inverse hysteresis effect observed in the current study. As a full-body movement involves larger muscle groups than a reaching movement, the associated, high mechanical costs might be sufficient to induce a negative PoR. Inverse hysteresis has previously been described for children reaching for cubes of in- or decreasing sizes (62, 63). Here, the point-of-change (PoC) between using one or two arms shifted as a function of direction. The mean PoC shift across children supported a hysteresis effect, but the authors found a large behavioral variance (63), and a small subset of the children exhibited an inverse effect.

A secondary question of the current study was whether full-body planning tasks could be conducted more efficiently in VR without reducing scientific validity. The general pattern of rotation angles in the real and virtual environment was similar (Figure 2A), but the critical *A*/*X* ratio was higher in the virtual environment, indicated by a significant main effect of “environment”. This is in line with previous studies, which reported similar kinematics in both environments (46, 47) but a more cautious behavior in VR (48, 49). Gérin-Lajoie et al. (52) specifically measured the personal space (PS; distance participants maintained from an obstacle) in a collision avoidance task. The authors found that PS shape was similar in a real and virtual environment, but PS size was larger in VR. Thus, if participants in our study sought to maintain a larger PS in the virtual environment, they had to initiate rotations at a higher *A*/*X* ratio.

A potential explanation for the larger PS in the virtual environment might be a larger movement variance. For arm movements, an increase in variance in the virtual environment has been found in two studies (47, 49). Horsak et al. (44) measured gait kinematics on a treadmill and found a larger kinematic variability in the virtual environment, as well as an increase in double support time, indicating more cautious behavior in VR. The results of our analysis of posture variance therefore are in line with the literature: Posture variance was significantly higher in the virtual environment (Figure 4A). To pass the aperture successfully, our participants were not allowed to knock over the slalom poles. Therefore, the increase in critical *A*/*X* ratio might reflect a response to the increase in movement variability.

An increase in variability could also explain the main effect of “fatigue” found in the virtual environment. Muscle fatigue leads to increased muscle activation, which manifests as an increase in EMG amplitude (71). According to Harris and Wolpert (72), signal noise in the motor system increases with signal amplitude, thereby increasing the movement variance. Therefore, the shift to a larger critical *A*/*X* ratio in the later trials of the current study might reflect participants' response to an increased movement variability due to muscle fatigue. Unfortunately, we were unable to test this with our current data as the factor “fatigue” had to be aggregated for the variance analysis. The fact that the main effect of “fatigue” was only significant in the virtual environment may be due to participants executing a larger number of trials in Experiment 2. The effect of “fatigue” was also visible in the combined analysis and shifted the critical *A*/*X* ratio into the same direction (cf. Table 2, Table 3; Supplementary Figures S1E,F), but it did not reach significance.

A similar increase in critical ratio with increasing movement variability has also been observed in other full-body posture selection studies: Van der Meer (34) asked participants to pass below a horizontal barrier, either with a straight or ducked posture. The authors found that the critical barrier-to-height (*B*/*H*) ratio significantly increased when participants switched from walking to running. In the original aperture task, Warren and Whang (38) also observed an increase in the critical *A*/*S* ratio when switching from walking to running, albeit not large enough to reach significance. As a reviewer pointed out, the increase in critical *A*/*S* ratio could also reflect a practice effect, that is, participants becoming more familiar with the virtual environment (or the task in general). While we cannot rule out this possibility based on the current data, the pattern of results at least suggests otherwise, as a practice effect should theoretically result in a less cautious (or more efficient) movement. However, the increase in critical ratio with an increasing number of trials found in the current study suggests the opposite.

In the virtual environment, we also tested if a cost-intensive full-body tracking and visualization was necessary to reproduce the findings of a real environment or if simple head-tracking would suffice. A significant main effect of “body visible” suggested that behavior depended on the visual feedback. Surprisingly, the critical *A*/*X* ratio was lower without visual feedback and, thus, more similar to the real environment (Figure 3A): Participants were less cautious when passing through the aperture. This might be due to a lack of error feedback in trials without visual feedback. With visual feedback, in contrast, participants could knock over the slalom poles and fail the task. One may hypothesize that this led to the more cautious behavior found in the “body visible” condition. Despite the significant difference, it should be noted that the effect of visual feedback was “small”.

Further, the findings for the posture variance did not align with a more cautious behavior, as variance was (slightly) higher when visual feedback was available (Figure 4C). Therefore, it is also possible that the visual feedback resulted in a more proximal, internal focus. This, in turn, may have reduced the automaticity of movement planning and increased muscle activity and posture variance (73). Importantly, there was no interaction of “order” and “body visible”. Presuming sufficient power, this finding indicates that the inverse hysteresis effect found in the current study was not affected by the visual feedback.

Overall, our comparison of the behavior in the real and virtual environment yielded mixed results. On the one hand, the pattern of results was qualitatively similar in the real and virtual environment, and with and without visual feedback of the own body. More importantly, the inverse hysteresis effect that we did not even expect before the experiment was faithfully reproduced in all conditions. Based on these findings, one could argue that even a simple VR system with head-tracking only would suffice to conduct ecologically valid full-body posture selection experiments in the future. Thus, space, equipment, and personnel requirements could be significantly reduced. On the other hand, we found a significant shift of the critical *A*/*X* ratio and a significant difference in posture variance, both with a large effect size, between environments. This may be due to a higher movement variance in VR resulting in a more cautious behavior in the virtual environment. Therefore, a validation of critical findings from VR studies in a real environment still seems advisable.

An interesting finding of the current study was the non-significant interaction between “condition” and “order”. The size and direction of the inverse hysteresis effect did not differ significantly between the randomized and ordered condition. This contrasts with most previous studies on posture (3, 10) and path selection (21) who tested for an effect of condition: In these studies, hysteresis was significantly reduced in a randomized sequence of trials. On the one hand, this could be due to the considerably smaller effect size of inverse hysteresis compared to the large effect size found for reaching (2). On the other hand, another study (on path planning in a center-out reaching task) also found that hysteresis effects did not depend on the predictability of the sequence (18).

It should be noted that, even within reaching movements, findings from one domain do not necessarily apply to the other: In addition to the differences observed for predictability mentioned above, a hand switch has been shown to cancel hysteresis in posture planning (74) but not in path planning (20). In path planning, hysteresis decays within 1,000–1,500 ms (19, 21) but persists for several seconds in posture selection (4). Therefore, the insensitivity of the inverse hysteresis effect to a change in predictability could be attributed to the switch from a reaching to a full-body posture selection task. Alternatively, inverse hysteresis might depend on completely different influencing factors than hysteresis. This needs to be a subject of future studies.

Another unexpected result was the (slightly) larger posture variance in the ordered condition of the combined analysis. To our best knowledge, no other full-body study to date has measured variance in both ordered and randomized tasks. In a reaching task, Schütz and Schack (7) showed similar variances for the randomized and ordered condition, when sequences in the ordered condition were split by “order”. Warburton et al. (21) predicted similar variances in path selection for both conditions using their reinforcement learning model. Thus, our finding contrasts with the (sparse) literature. However, it should be noted that the effect from the combined analysis could not be reproduced in the second analysis, and that the effect size was “very small”. Therefore, it may be a coincidental finding.

In summary, in the current study, we asked whether hysteresis was a general motor planning principle rooted in the cognitive system, that not only applies to reaching movements but to full-body movement as well. To this end, we used a full-body posture selection task: passing through apertures of different widths. We replicated the task in a virtual environment to test the feasibility of using VR to investigate full-body movements. Our findings showed a significant inverse instead of a normal hysteresis effect. This supports the notion that repetition suppression, which to date has primarily been described in perception research, also applies to motor planning. The inverse effect may stem from the high mechanical costs of full-body movements. The effect was replicated in a second group of participants in a virtual environment, demonstrating the feasibility of using VR for these types of experiments.

## Data Availability

The raw data supporting the conclusions of this article will be made available by the authors, without undue reservation.
