# Supplementary material for: Inverse hysteresis effects in a real and virtual environment
Source: Front Sports Act Living. 2026 Feb 25;8:1734971. doi: 10.3389/fspor.2026.1734971 (PMC12977077; doi:10.3389/fspor.2026.1734971)
Supplement: Supplementary file 1 [file Image1.pdf]

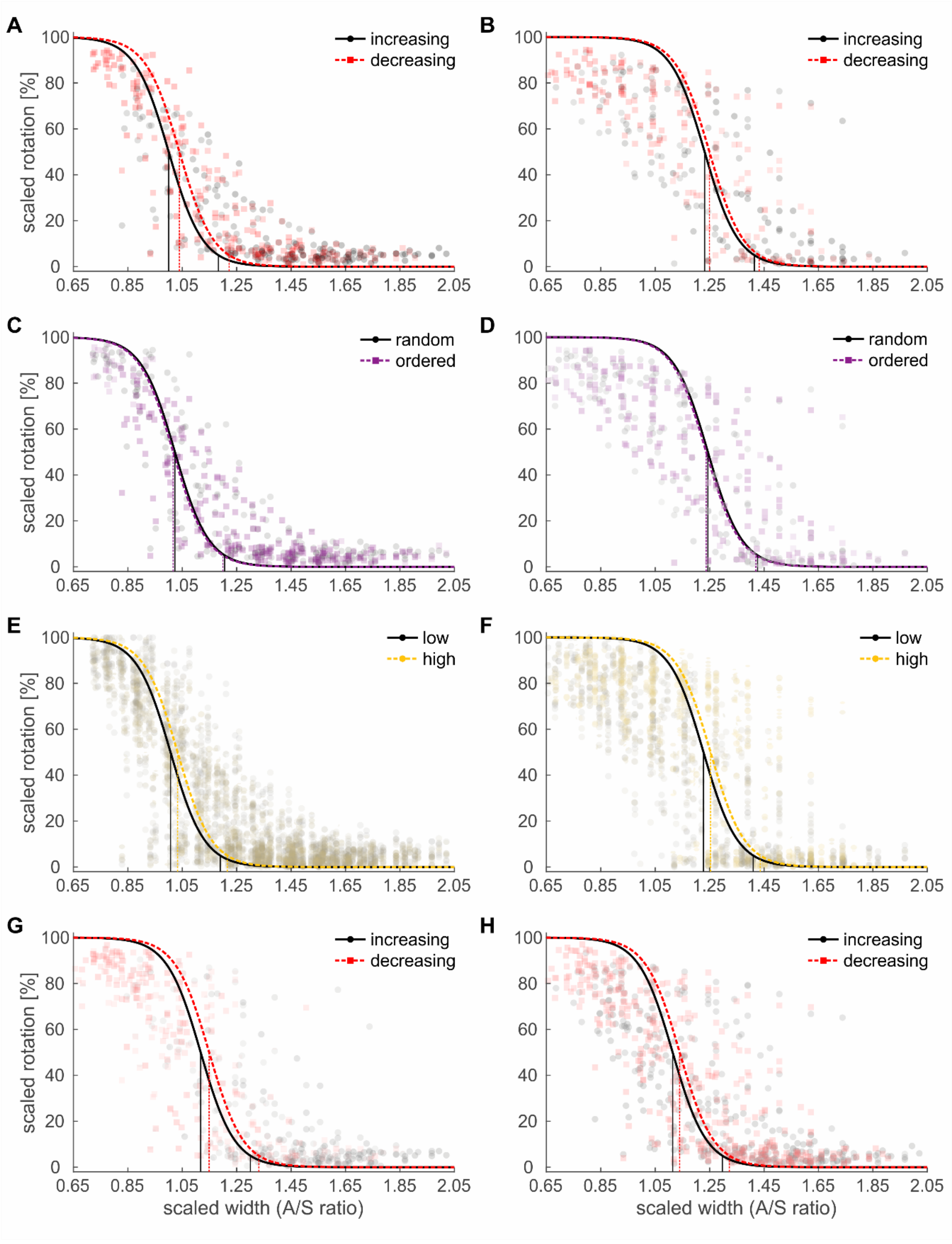

Fig S1 Scaled shoulder rotation, plotted against 'A/S ratio', A,B,G,H split by 'order', **C,D** split by 'condition', **E,F** split by 'fatigue'. Panels **A,C,E** real 'environment', panels **B,D,F** virtual 'environment', panel **G** randomized 'condition', panel **H** ordered 'condition'. *Markers* show the average rotation values of the participants at each of their 9 individual A/S ratios. *Thick lines* show the logistic curves of the fixed effects of the GLMM. *Thin, vertical lines* mark the points-of-change (50% rotation) and critical values (5% rotation). *Notes: # of data points included in each marker is reflected by its face opacity. Face opacity in panels E,F was increased by a factor of 2 (in comparison to the other panels) to render the data points more visible. Interaction between 'environment' and 'order' in panels A,B. Interaction between 'condition' and 'order' in panels G,H*
